# Genomic signatures of bottleneck and founder effects in dingoes

**DOI:** 10.1002/ece3.10525

**Published:** 2023-09-19

**Authors:** Manoharan Kumar, Gabriel Conroy, Steven Ogbourne, Kylie Cairns, Liesbeth Borburgh, Sankar Subramanian

**Affiliations:** ^1^ School of Science, Technology, and Engineering The University of the Sunshine Coast Moreton Bay Queensland Australia; ^2^ Centre for Bioinnovation The University of the Sunshine Coast Sippy Downs Queensland Australia; ^3^ School of Science, Technology, and Engineering The University of the Sunshine Coast Sippy Downs Queensland Australia; ^4^ Evolution and Ecology Research Centre, School of Biological, Earth and Environmental Sciences UNSW Australia Sydney New South Wales Australia; ^5^ Centre for Ecosystem Science, School of Biological, Earth and Environmental Sciences UNSW Australia Sydney New South Wales Australia

**Keywords:** deleterious SNVs, dingo, founder effect, inbreeding, mutation load, population bottleneck, small populations

## Abstract

Dingoes arrived in Australia during the mid‐Holocene and are the top‐order terrestrial predator on the continent. Although dingoes subsequently spread across the continent, the initial founding population(s) could have been small. We investigated this hypothesis by sequencing the whole genomes of three dingoes and also obtaining the genome data from nine additional dingoes and 56 canines, including wolves, village dogs and breed dogs, and examined the signatures of bottlenecks and founder effects. We found that the nucleotide diversity of dingoes was low, 36% less than highly inbred breed dogs and 3.3 times lower than wolves. The number of runs of homozygosity (RoH) segments in dingoes was 1.6–4.7 times higher than in other canines. While examining deleterious mutational load, we observed that dingoes carried elevated ratios of nonsynonymous‐to‐synonymous diversities, significantly higher numbers of homozygous deleterious Single Nucleotide Variants (SNVs), and increased numbers of loss of function SNVs, compared to breed dogs, village dogs, and wolves. Our findings can be explained by bottlenecks and founder effects during the establishment of dingoes in mainland Australia. These findings highlight the need for conservation‐based management of dingoes and the need for wildlife managers to be cognisant of these findings when considering the use of lethal control measures across the landscape.

## Graphical abstract

Dingoes arrived in Australia during the mid‐Holocene. Although dingoes subsequently spread across the continent, the initial founding population(s) could have been small. We investigated this hypothesis by comparing the whole genomes of dingoes and other canines. We found that the nucleotide diversity of dingoes was lower, the number of runs of homozygosity (RoH) segments was higher and the numbers of homozygous deleterious Single Nucleotide Variants (SNVs), and loss of function SNVs were higher than those observed for breed dogs, village dogs, and wolves. Our findings can be explained by bottlenecks and founder effects during the establishment of dingoes in mainland Australia.

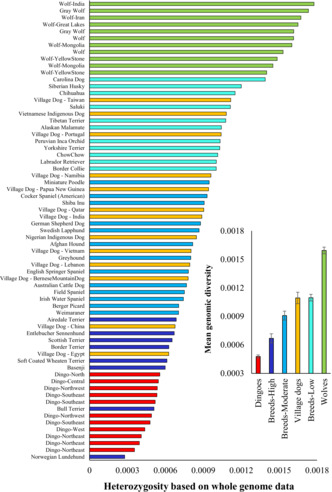

## 1 INTRODUCTION

Dingoes arrived in Australia between 4000 and 11,000 years ago, and their ancestors probably originated in Asia (Balme et al., 2018; Bergstrom et al., 2020; Cairns & Wilton, 2016; Savolainen et al., 2004; Zhang et al., 2020). While their taxonomic status and evolutionary history remain contested, studies demonstrate their important ecological role as Australia's largest terrestrial top‐order predator (Johnson et al., 2007; Letnic et al., 2012; Wallach et al., 2010) and also their cultural significance, particularly for First Nations Peoples (Archer‐Lean et al., 2015; Corbett, 2001). Currently, dingo populations are found across the entire continent (excluding Tasmania), occupying vastly different habitats ranging from the mountainous Australian Alpine regions, tropical, subtropical, and temperate regions, the arid zones of Australia and offshore coastal barrier islands such as K'gari (Fraser Island). Dingo populations can vary widely in phenotypic features, such as body weight, coat colour, and social behaviour (Cairns et al., 2021; Corbett, 2001; Koungoulos, 2020; Tatler et al., 2020). Despite their continent‐wide presence and array of phenotypic variation, the size of the founding population of dingoes is likely to be small, given Australia's geographic isolation, the narrow window for their arrival, and the potential that they were transported by humans. With the advent of cost‐effective sequencing technologies, it is now possible to obtain the whole genome sequences and examine the genetic signatures of founder effects in the dingoes.

The nucleotide diversity of a population is determined by mutation rate (*μ*) and effective population size (*N*_e_) (Hartl, 2007). Since the *μ* of a species is fairly constant over time, the reduction in nucleotide diversity is dictated by *N*_e_ (Kimura, 1983). Therefore, measuring nucleotide diversity using whole genome data is a simple measure to detect the founder effects. The use of whole genome data also enables the measurement of lengths of long tracts of homozygous genotypes or runs of homozygosity (RoH) segments (Ceballos et al., 2018). A number of previous studies showed that bottleneck and founder effects increase the number of long RoHs (Bortoluzzi et al., 2020; Khan et al., 2021; Sánchez‐Barreiro et al., 2021; Tournebize et al., 2022). Hence, estimating the lengths of RoH in dingoes and comparing them with those observed for other canines will potentially reveal whether the size of the founding dingo population was small. The third hallmark of the founder effect is the accumulation of deleterious genetic variations (Evans, 2015; Livingstone, 1969). The ratio of diversities at nonsynonymous and synonymous sites is routinely used to measure the deleterious mutational load in populations (Lohmueller et al., 2008; Makino et al., 2018; Marsden et al., 2016; Subramanian, 2016). These studies observed a much higher ratio in small populations than those in large populations, and the high ratio suggests a higher proportion of nonsynonymous Single Nucleotide Variants (SNVs). Reduction in population size was also known to increase the frequencies of rare deleterious variants, and hence the number of homozygous SNVs is expected to be high in bottlenecked populations. Indeed, significantly higher numbers of homozygous nonsynonymous SNVs are frequently reported in small or bottlenecked populations (Marsden et al., 2016; Subramanian, 2021; Xie et al., 2018). Studies of bottlenecked populations also observe an abundance of homozygous SNVs that were predicted to be deleterious based on their functional consequences or evolutionary constraints. Therefore, the founder effect on dingo populations could be investigated by using whole genome data and measures that quantify the magnitude of the accumulation of deleterious variants in their genomes. Such analyses could provide insight into management concerns for these ecologically and culturally significant dingo populations that currently suffer from persecution in most Australian jurisdictions and are subject to widespread lethal control measures.

In our study, we investigated the genomic evidence for founder effects and bottlenecks in dingo populations by sequencing the whole genomes of three dingoes and obtaining the genome data of nine additional dingoes from mainland Australia. We also obtained genome data of breed dogs, village dogs, and wolves to give context to our analysis. We performed comparative analyses of these canine genomes and estimated various measures to capture the genetic diversity, runs of homozygosity, and accumulation of deleterious genetic variants.

## 2 MATERIALS AND METHODS

### 2.1 Samples and whole‐genome sequencing

The saliva samples from three dingoes were collected: one from Kimberley in Western Australia, one from western New South Wales, and a captive individual from the Alpine region of Southeast Australia. The samples were shipped to Novogene Co., Ltd., for DNA extraction and sequencing. DNA extraction was performed by following standard operating protocols using saliva/tissue extraction kits. The library building and sequencing were performed following Illumina standard protocols. The genomic DNA was randomly sonicated into short fragments. Later, these fragments were end‐repaired, added with A‐Tail in the end, and ligated with an Illumina adapter. Then the fragments only with adapters were PCR amplified, size selected, and purified for short read sequencing. Furthermore, the purified library was checked with Qubit and real‐time PCR for quantification and a bioanalyzer for size distribution detection. Quantified libraries were sequenced on the Hiseq 2000 Illumina platforms, according to effective library concentration, to obtain 30 times coverage with 150 base pair paired‐end reads. The analysis based on the sequence reads suggested that the coverage was 8X–10X (Table S2). The reduction in the coverage of dingo genomes could be attributed to the presence of bacterial DNA in the saliva samples.

### 2.2 Whole‐genome data collection

In addition to the three samples from this study, we obtained the raw sequence reads in *fastq* format for nine mainland dingoes from a previous study (Zhang et al., 2020). The 12 samples come from the North (1), Northwest (2), Northeast (3), West (1), Central (1), and Southeast (4) regions of the Australian continent (see Figure S1 and Tables S1 and S2). We excluded one dingo genome from K'gari (Fraser Island) reported by Zhang et al. (2020). A previous study showed a significant reduction in the heterozygosity of the K'gari‐Fraser Island dingo population compared to the mainland dingoes (Conroy et al., 2021). Therefore, we excluded the Island dingo in order to avoid the confounding effect of an additional bottleneck that could have occurred when the subset(s) of mainland dingoes became island‐bound. In addition to dingoes, we obtained whole genome *fastq* data from 11 wolves, 13 village dogs, and 32 breed dogs (see Table S2) from another study (Plassais et al., 2019). The 32 dog breeds were chosen based on two criteria. First, we obtained dog breeds representing various geographical locations, including the Arctic, African, modern European, Asian and Middle Eastern regions. The geographic locations of the breeds also reflect their distinct clades in the canine phylogeny (Vonholdt et al., 2010). Second, out of 722 breed dogs from the previous study (Plassais et al., 2019), we selected 10 breeds having very high inbreeding coefficients. Since a very small number of founding individuals might have been used during breed formation, these breeds are the best candidates for comparison with dingoes. We also included the coyote genome obtained from the same study (Plassais et al., 2019) to use it as the outgroup. The *fastq* sequences of these genomes were downloaded from the NCBI SRA database (https://www.ncbi.nlm.nih.gov/sra). Relevant SRA ID details can be found in (Table S2) supplementary information. These fastq reads were processed along with the newly sequenced three dingo samples as described below.

### 2.3 Bioinformatics analyses

Illumina raw reads of three dingo samples were trimmed based on quality and adapter sequences using Trimmomatics‐0.36 (Bolger et al., 2014) (options: ILLUMINACLIP: adapter.fa:2:30:10 SLIDINGWINDOW:4:20 MINLEN:50). Similarly, we processed fastq reads of 68 samples of dingoes (11) and other canines (56) plus coyote obtained from published studies (Plassais et al., 2019; Zhang et al., 2020). Then, each sample's clean reads were aligned to the canfam3 (https://hgdownload.soe.ucsc.edu/goldenPath/canFam3/bigZips/canFam3.fa.gz) reference genome using the bwa tool (Li & Durbin, 2009) (options: bwa mem‐M). Mapped reads in the sequencing alignment mapped (SAM) format were converted to binary alignment/mapped (BAM) format using samtools version 1.31 (options: samtools view ‐@ 8 ‐b ‐F 4). Then, we sorted the aligned reads based on chromosomes in BAM format. The sorted reads were used for marking PCR duplicates using the Picard tool. Later, we generated genotypes for all sites using samtools (Li, 2011) (options: samtools mpileup ‐C 50 ‐uf | bcftools call ‐c ‐V indels ‐O z). Finally, we merged all position genotypes of 68 genomes using bcftools merge (par: ‐m all). After merging, we filtered biallelic variant sites using an in‐house awk script. The final dataset had a total of 29,153,123 biallelic positions.

To identify synonymous and nonsynonymous sites of the protein‐coding genes we used the software *SNPeffect* tool (De Baets et al., 2012) (options: java ‐jar snpEff.jar eff CanFam3) using the CanFam3.1 annotations obtained from the .gff file (https://ftp.ncbi.nlm.nih.gov/genomes/all/annotation_releases/9615/105/GCF_000002285.3_CanFam3.1/GCF_000002285.3_CanFam3.1_genomic.gff). In addition, we estimated the average read depth for each sample using the “depth” module of the software *samtool*. The total number of sites covered was calculated for each sample using an in‐house script. The number of runs of homozygosity segments was estimated using the software *plink* (Purcell et al., 2007) with the following parameter (‐‐geno 0.01 ‐‐homozyg ‐‐homozyg‐window‐het 0 ‐‐maf 0.05). To identify the ancestral state of the variants, we used the whole genome data of coyote to determine the direction of mutational change and to identify the derived alleles.

### 2.4 Population genetics and statistical analyses

The software *plink* (Purcell et al., 2007) was used to perform Principal Component Analysis (PCA). The Maximum Likelihood‐based method *Admixture* (Alexander et al., 2009) was used to determine the admixture patterns of canine genomes. While we observed the lowest cross‐validation error for *K* = 2 (two ancestries), the canine populations were not separated at this level. Therefore, we used *K* = 4 to show the admixture patterns among canines as it separated the ancestries of dingoes, village dogs, breed dogs, and wolves. Higher *K* values (5 and 6) did not split the dingo populations further (but divided the breed dogs), suggesting more homogeneity among the dingoes used in this study. The inbreeding coefficients were calculated for the breed dogs using *plink*. This revealed a large variation in these estimates among breeds (0.12–0.92). Therefore, any mean estimate computed for breed dogs as one group will have confounding effects. To remove this bias, we grouped breed dogs into three categories based on their inbreeding coefficients. The high, moderate, and low inbred categories consist of breeds having inbreeding coefficient estimates of >0.4 (12), 0.4–0.2 (13), and <0.2 (6), respectively. To identify deleterious missense alleles, we first used the *SIFT* score (Ng & Henikoff, 2003), which determines the deleteriousness of a variant based on the level of evolutionary constraints on genomic positions. Based on this method, we considered the SNVs present on genomic positions with a SIFT score ≤0.05 as ‘deleterious’. Next, we used the functional consequences of an SNV to determine its deleteriousness. If an SNV results in premature termination of transcription or translation, it is considered as ‘deleterious’. These Loss of function (LoF) SNVs were identified based on the annotations “stop_lost”, “stop_gained”, “start_lost”, “splice_donor” and “splice_acceptor”. The numbers of synonymous and nonsynonymous SNVs were divided by their respective number of synonymous and nonsynonymous sites to obtain the diversities at these sites. The ratio of the two was used to determine the strength of purifying selection or the accumulation of deleterious nonsynonymous SNVs in each genome. The average number of LoF and deleterious SNVs per genome, along with the standard errors, were estimated for each genome. We also obtained the dingo reference genome (ASM325472v2) and using the program *liftover* we gathered the chromosomal coordinates and reference alleles of dingo for each camfam3 position. The numbers of nonsynonymous and deleterious SNVs were then calculated using the dingo reference as well.

### 2.5 Statistical analyses

To estimate the significance of the mean estimates between the groups of canines we used the *Z* test. For this purpose, we estimated the normal deviate (*Z*) between any two groups and obtained the *p* values using the software *Z* to *P* (http://vassarstats.net/tabs_z.html). A Pearson correlation coefficient was used to determine the strength of the correlation. Furthermore, using the non‐parametrical Spearman's correlation also produced a similar strength of correlation. The statistical significance of the correlation was determined by converting the correlation coefficient *r* to the normal deviation *Z*, and this was accomplished using the online software *r* to *P* (http://vassarstats.net/tabs_r.html).

## 3 RESULTS

### 3.1 Nucleotide diversity based on the whole genomes of dingoes and other canines

**FIGURE 1:**
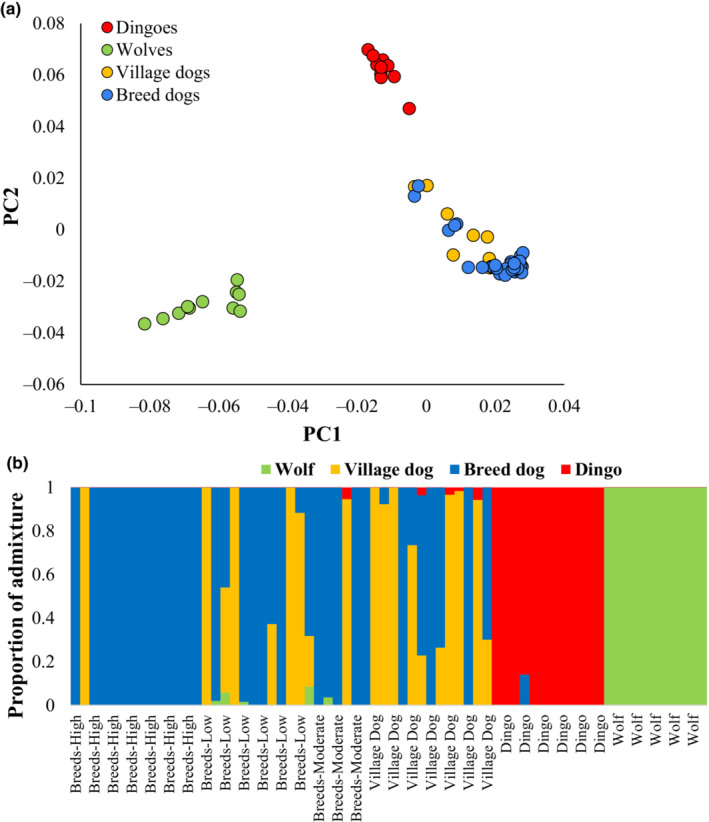
(a) The scatter graph showing the results from the principal component analysis (PCA) using the whole genomes of dogs, dingoes, and wolves. (b) The proportion of genetic admixture in each canine genome is shown. This analysis was performed assuming four ancestries for canines (*K* = 4).

**FIGURE 2:**
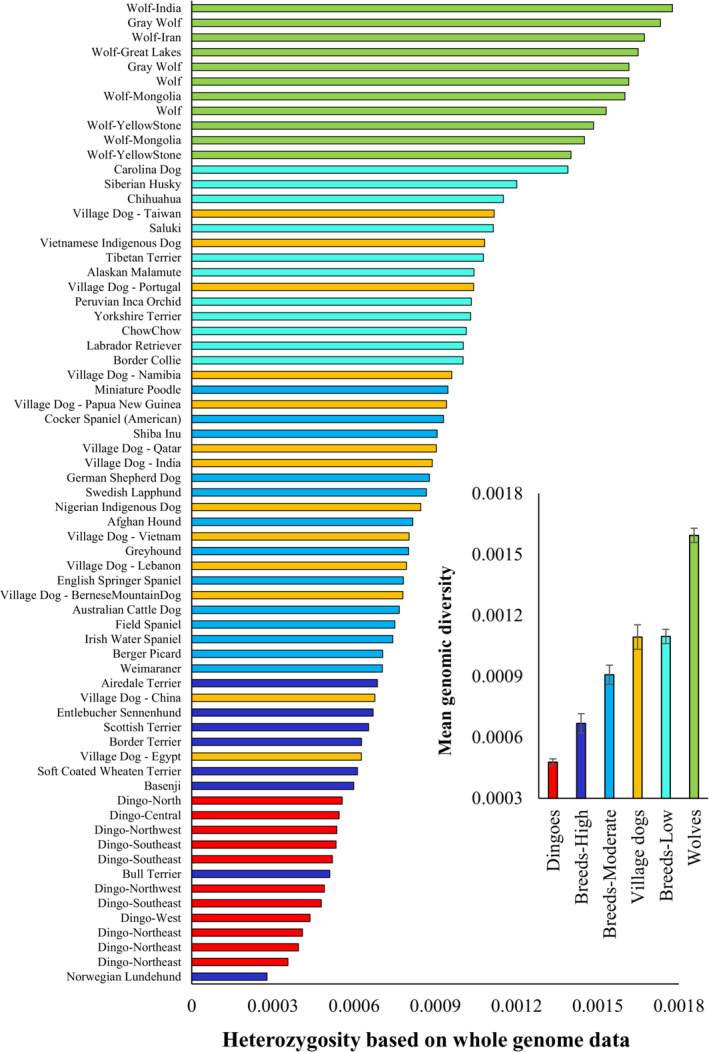
Heterozygosity or nucleotide diversity estimates for dingoes and other canines. Nucleotide diversity was estimated using the whole genome sequences. The dog breeds were grouped into three categories based on their inbreeding coefficients – Highly inbred (>0.4), Moderately inbred (0.4–0.2), and lowly inbred (<0.2). *Inset*: Average nucleotide diversities computed for various canine groups. Error bars show the standard error of the mean.

In this study, we sequenced three dingo genomes and assembled the whole genome dataset from previously published dingoes, dog breeds, village dogs, and wolves (see Table S1). Using this data, we performed principal component analysis to determine the level of population structure among the canines. This revealed three distinct clusters of dingoes, dogs, and wolves (Figure 1a). To investigate the genetic admixture, particularly for dingoes, we conducted an admixture analysis (Figure 1b). This showed one individual from Southeastern Australia had a small amount of admixture (14%) from dogs, which suggests that this may not be a ‘pure’ or unadmixed dingo. Therefore, we excluded this dingo from further analysis and estimated the heterozygosity or nucleotide diversity using the whole nuclear genomes of 11 dingoes and other canines. We found dingoes to have the lowest nucleotide diversities compared to all other canines analysed, except for Norwegian Lunde Hund and Bull terrier dog breeds (Figure 2). We then grouped breed dogs into three categories based on their inbreeding coefficients (see 2. Materials and methods). The mean diversity estimate of dingoes was significantly lower (*p* < .0001) than that of other canine groups (Figure 2, inset). For instance, the nucleotide diversity of dingoes was 36% less than that of the highly inbred breed dogs. The mean estimates of the three dog breed groups (high, moderate, and low) vary significantly, and the diversity of village dogs was less than that of the low‐inbred group. The mean diversity of wolves was found to be the highest of all canine groups, and it was 3.3 times higher than observed for dingoes.

### 3.2 Runs of homozygosity

**FIGURE 3:**
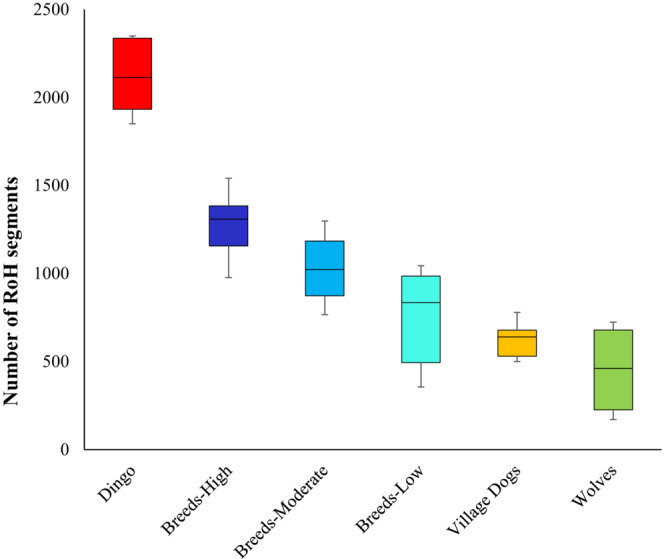
Box plot showing the mean estimates of the number of runs of homozygosity (RoH) segments for dingoes, dog breeds, village dogs, and wolves. A threshold of 0.5 Mb or more was used to determine an RoH segment. The crosses indicate means, and the line within the box denotes the median. The whiskers show the maximum and minimum values.

Using the whole genome data, we next estimated the number of runs of homozygosity (RoH) segments. For this analysis, we used a threshold of 0.2 Mb to define an RoH segment, and we also used a stringent criterion of not allowing any heterozygous SNVs within an RoH. This analysis revealed that dingoes have a much higher number of RoH segments than any other canine group (Figure 3). The mean number of RoH segments in dingoes was 2125, which was 62% higher than that estimated for the highly inbred breed dogs (1313), and the difference between the estimates was highly significant (*p* < .0001). The number of RoH segments per genome estimated for breed groups varied 1.6 times as the high and low inbred groups had 1313 and 814 segments, respectively. The average numbers of RoH segments in village dogs (605) and wolves (451) were four times smaller than that of dingoes.

### 3.3 Deleterious mutational load in dingoes and other canines

**FIGURE 4:**
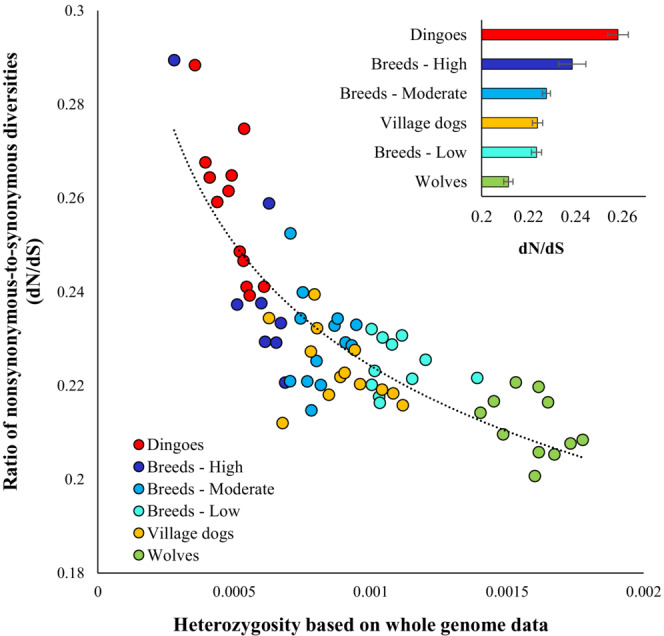
Correlation between heterozygosity estimates based on whole genomes and the ratio of nonsynonymous‐to‐synonymous diversities (dN/dS). The relationship is highly significant (*r* = 0.92 and *p* < .00001). A regression analysis was used, and the best‐fitting Power Law (log–log) curve is shown. *Inset*: Average dN/dS computed for various canine groups. Error bars show the standard error of the mean.

To measure the accumulation of deleterious mutational load in canine genomes, we first estimated diversity at nonsynonymous (dN) and synonymous (dS) sites after identifying the protein‐coding genes using genome annotations. The ratios (dN/dS) of the two estimates were then calculated, and those were plotted against the nucleotide diversities obtained for each genome. This analysis showed a highly significant negative correlation (*r* = 0.92, *p* < .000001) between the two variables (Figure 4). Importantly, the dN/dS ratios of dingoes were predominantly higher than those of other canines, and the mean dN/dS estimate of dingoes (0.27) was significantly higher than that of highly inbred breed dogs (0.24) and other groups (*p* < .0001; Figure 4, inset).

**FIGURE 5:**
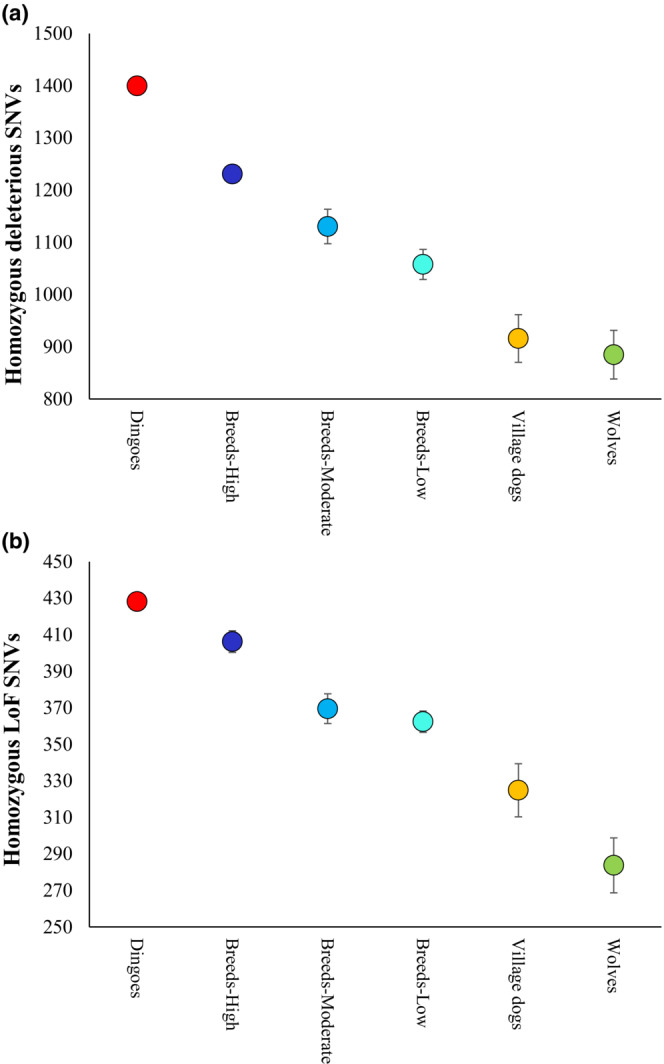
Mean counts of (a) homozygous deleterious single nucleotide variants (SNVs) and (b) homozygous Loss of Function (LoF) SNVs computed for the canine genomes are shown. The average estimates of dingoes were statistically different from those of the highly inbred breeds and other groups (*p* < .0001). Error bars show the standard error of the mean.

**FIGURE 6:**
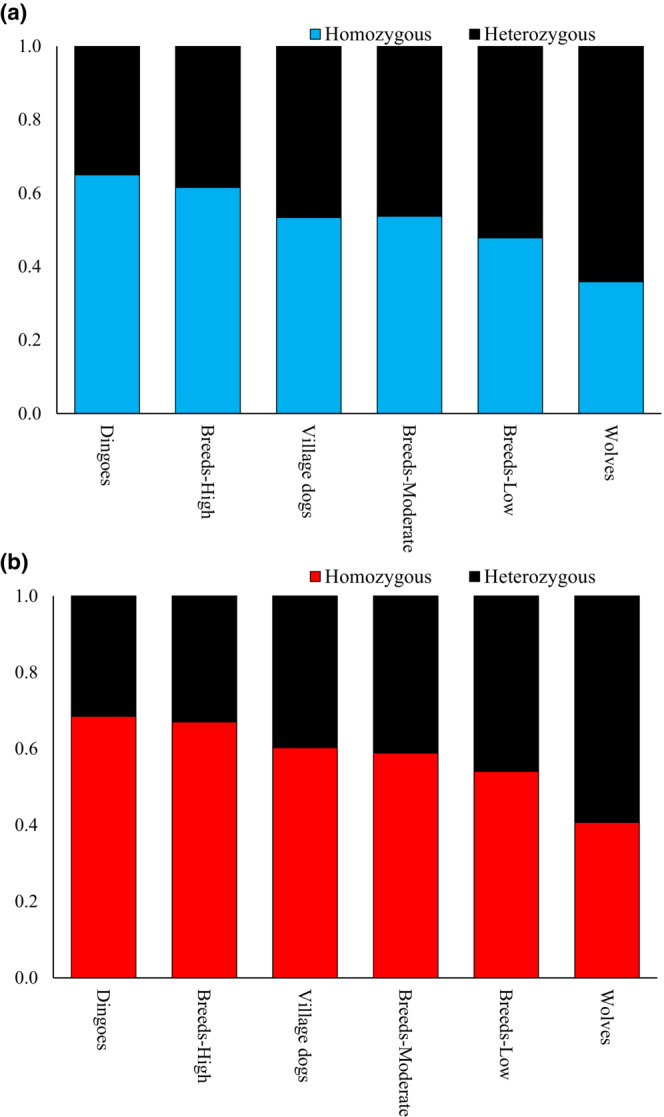
The stacked columns show the proportion of homozygous and heterozygous single nucleotide variants (SNVs) estimated for the canine genomes. (a) Deleterious SNVs (b) Loss of function SNVs.

To measure the load of deleterious SNVs, we employed two methods. First, we used the SIFT scores to identify the deleterious nonsynonymous SNVs present in evolutionarily constrained positions. Second, the functional annotations were used to detect the highly deleterious LoF SNVs that cause premature termination of protein synthesis. Our analyses identified that dingoes had the highest load of homozygous deleterious missense SNVs (Figure 5a) and LoF SNVs (Figure 5b) of all the canines studied. We observed that dingo genomes harboured an average of 1394 homozygous deleterious missense SNVs and 435 homozygous LoF SNVs, which were significantly (*p* < .0001) higher than those estimated for the highly inbred breed dogs (1239 and 406, respectively) and other canine groups. Wolves had the lowest number of homozygous deleterious missense SNVs (885) and homozygous LoF SNVs (283). We also plotted the proportions of homozygous and heterozygous deleterious (Figure 6a) and LoF SNVs (Figure 6b) in the canine genomes. This revealed that dingoes have the highest and wolves have the lowest proportion of homozygous SNVs. An opposite pattern was observed for the proportions of heterozygous SNVs in the canine genomes. To remove any confounding effects, we also re‐estimated the dN/dS ratios and counts of deleterious SNVs of dingoes using the dingo reference genome (see 2. Materials and methods). The results of these analyses were almost identical to those presented in Figures 4, 5, 6 (see Figures S2 and S3).

## 4 DISCUSSION

Using whole‐genome data, we examined the genomic signatures of dingoes, breed dogs, village dogs, and wolves. We found evidence for bottlenecks and founder effects in dingoes. Overall, despite the sixfold variation in heterozygosity or nucleotide diversity observed within canines (0.0003–0.0018), dingoes had the lowest diversity of all the canines studied (Figure 1). The variation in nucleotide diversity suggests that different canine populations may have large differences in effective population sizes. The mean diversity of dingoes was 36% less than that of highly inbred breed dogs and ~4 times lower than that of wolves. The observed low diversity in dingoes corroborates previous reports using a small number of breeds (Freedman et al., 2014; Zhang et al., 2020). These findings have important implications for the conservation of dingoes but also for understanding their evolutionary history and relationship to other canines.

Previous studies showed that the dN/dS ratios for domesticated animals (such as dogs, cows, yak, pigs, and silkworm) and plants (such as rice, soybean, and sunflower) were significantly higher than those of their wild counterparts (Bosse et al., 2019; Dussex et al., 2021; Kono et al., 2016; Lu et al., 2006; Makino et al., 2018; Marsden et al., 2016; Mezmouk & Ross‐Ibarra, 2014; Pedersen et al., 2017; Peischl et al., 2018; Ramu et al., 2017; Renaut & Rieseberg, 2015; Robinson et al., 2019; Subramanian, 2021; Xie et al., 2018). Furthermore, domesticated plants and animals were found to have more homozygous deleterious SNVs than their wild relatives (Marsden et al., 2016; Subramanian, 2021; Xie et al., 2018). In contrast, our results revealed the opposite: a much higher dN/dS ratio and more homozygous deleterious SNVs in wild dingoes than the domesticated breed dogs. Although dingoes have been in the wild for over thousands of years, their initial founder population must have been small. Theories predict that selection is not strong enough to remove deleterious mutations because the effect of genetic drift is high in small populations. A similar observation was reported for the Przewalski horse population, which was almost extinct in 1969, and the ~2100 animals living now were derived from a very small captive stock of 12–15 animals (Orlando & Librado, 2019). Genome analyses revealed a high dN/dS ratio and an elevated proportion of homozygous deleterious SNVs in this population.

Contemporary dingo populations are found across the entire continent, and hence the rate of historical inbreeding can be presumed to be low (due to the ongoing lethal control programs, contemporary populations in some regions are becoming small). Therefore, the observed low diversity and high load of deleterious mutations in dingoes could be due to a limited number of individuals in the initial founding populations. In contrast, the diversity and mutation load of breed dogs were modulated by intense inbreeding and artificial selection. To disentangle the bottleneck/founder effect from inbreeding, we examined the length of RoH segments in dingoes and dog breed genomes. Figure 3 shows the number of RoH that are >0.2 Mb. However, we also estimated the number of very long RoH that are >2 Mb. Overall, the dog breeds had 67 very long RoH segments, and dingoes had only 52 of such segments per genome, and these estimates for highly (100 per genome) and moderately inbred breeds (57 per genome) were higher than that of dingoes. It is well known that inbreeding leads to a few very long RoH (>2 Mb), and in contrast founder effect or bottleneck results in a greater number of medium‐sized RoH (hundreds of Kbs) (Ceballos et al., 2018).

The results of this study could be influenced by the differences in the sequencing coverage between the genomes. To examine this, we plotted the coverage of the 722 dog breeds reported previously against the number of homozygous and heterozygous SNVs called (Figure S4). This analysis revealed that the number of SNVs identified increased with the sequence coverage only up to 7.5 times (7.5X) for homozygous (Figure S4a) and 15 times (15X) for heterozygous SNVs (Figure S4b). For any coverage above 7.5X, the number of homozygous SNVs identified remained the same. Similarly, the number of heterozygous SNVs called remained the same when the coverage was on or above 15X. Therefore, we excluded dingo and other canine genomes that have <15X coverage and reanalysedl the data. This filtering significantly reduced the number of genomes of dingoes, breed dogs and wolves, and all village dogs. However, despite this reduction, our results remained the same (Figures S5–S9). The mean estimates of nucleotide diversity (Figure S5), RoH (Figure S6), dN/dS ratio (Figure S7), deleterious (Figure S8), and Loss of function SNVs (Figure S9) obtained before and after filtration were very similar. The differences between the estimates obtained before and after the filtration were statistically not significant. Furthermore, we also showed these results by combining the breed dogs into one category (Figures S5B–S9B).

In this study, we found five lines of evidence for bottleneck and founder effects in dingo populations in Australia. First is the reduction in heterozygosity in dingo populations, and second is the presence of a large number of RoH segments in the genomes of dingoes. We used three different methods to show the accumulation of harmful mutations in dingoes – the high dN/dS ratio, elevated number of homozygous deleterious amino acid changing SNVs, and LoF SNVs in dingo populations compared to breed dogs, village dogs, and wolves. These findings could potentially be explained by an initial bottleneck, which might have been caused by a limited number of founders that served as the ancestral stock for the modern dingoes. Similar results have been reported in other populations. For instance, the reduction in the heterozygosity of human populations negatively correlates with the geographical distance from Africa (DeGiorgio et al., 2009). This is because when humans migrated out‐of‐Africa, only a small subpopulation served as the founders to the new location, and subsequent migrations from the new locations that were further away from Africa resulted in a serial founder effect (Henn et al., 2015). These founder effects are also reflected in the deleterious mutation loads of the population, which positively correlate with geographical distance from Africa (Henn et al., 2016). Similarly, populations that are far away from Africa also have a higher number of RoH than those in and close to Africa (Pemberton et al., 2012). Genomes of the populations that migrated recently, such as French Canadians, also showed the signatures of founder effects, including low diversity, a large number of RoH segments, and elevated deleterious mutation load (Roy‐Gagnon et al., 2011). Genetic signatures of founder effects have also been reported in many other species, such as Isle Royale wolves (Robinson et al., 2019), Corsican red deer (Hajji et al., 2008), Afognak Island elk (Hundertmark & Van Daele, 2010), and Common Myna (Hill & Pawley, 2018), Wrangel Island mammoth (Rogers & Slatkin, 2017), San Nicolas Island fox populations (Robinson et al., 2016) and Steward Island kakapo populations (Dussex et al., 2021).

The findings of this study help build our knowledge about the evolutionary history of dingoes and may have important implications for wildlife management. Our results highlight that despite their widespread distribution across Australia, the diversity of dingoes is still low compared to other canines, including the highly inbred dog breeds. We also found significant evidence of high accumulation of deleterious SNVs. Wildlife managers should begin considering the genomic consequences of lethal control programs on dingo populations. For example, aerial baiting programs which realise a 90% population knockdown (Ballard et al., 2018) may initiate further bottlenecks and exacerbate the low genetic diversity and high mutational loads of targeted dingo populations with detrimental consequences. For example, low nucleotide diversity and mutational loads in dingoes may increase the threat of diseases such as Ehrlichiosis, Parvo virus, Sarcoptic mange, and Canine distemper to dingo populations. Further study of the genomic health of dingo populations at regional and local scales may be prudent to inform conservation and management practices. Ongoing genetic monitoring of the genetic diversity of lethally controlled populations may assist managers in designing conservation‐aware dingo management plans, given the important ecological role and cultural significance of dingoes in the Australian landscape. The present investigation is based on a small number of dingoes, and hence there is a need for a wider study, including more genomes from various geographic locations. Such a study will also reveal any additional bottlenecks specific to different populations.

## AUTHOR CONTRIBUTIONS

**Sankar Subramanian:** Conceptualization (equal); formal analysis (equal); funding acquisition (equal); investigation (equal); project administration (equal); supervision (equal); writing – original draft (equal). **Manoharan Kumar:** Formal analysis (equal); software (equal); writing – review and editing (equal). **Gabriel Conroy:** Resources (equal); writing – review and editing (equal). **Steven Ogbourne:** Resources (equal); writing – review and editing (equal). **Kylie Cairns:** Resources (equal); writing – review and editing (equal). **Liesbeth Borburgh:** Data curation (equal).

## Supporting information


Data S1:

## Data Availability

The DNA sequence DNA of the three dingo genomes sequenced in this study are available in the SRA (Sequence Reads Archive) database under study accession PRJNA1004041. The SRA accession numbers of nine other dingoes and 56 other canine genomes used in this study are given in Table S2.
