# Supplementary material for: Genomic signatures of bottleneck and founder effects in dingoes
Source: Ecol Evol. 2023 Sep 19;13(9):e10525. doi: 10.1002/ece3.10525 (PMC10508967; doi:10.1002/ece3.10525)
Supplement: Supplementary file 1 — Data S1: [file ECE3-13-e10525-s001.pdf]

Genomic signatures of bottleneck and founder effect in dingoes

Manoharan Kumar[1], Gabriel Conroy[2], Steven Ogbourne[2], Kylie Cairns[3], Liesbeth Borburgh[2] and Sankar Subramanian[1*]

Supplementary Information

Figure S1: Locations of dingo samples

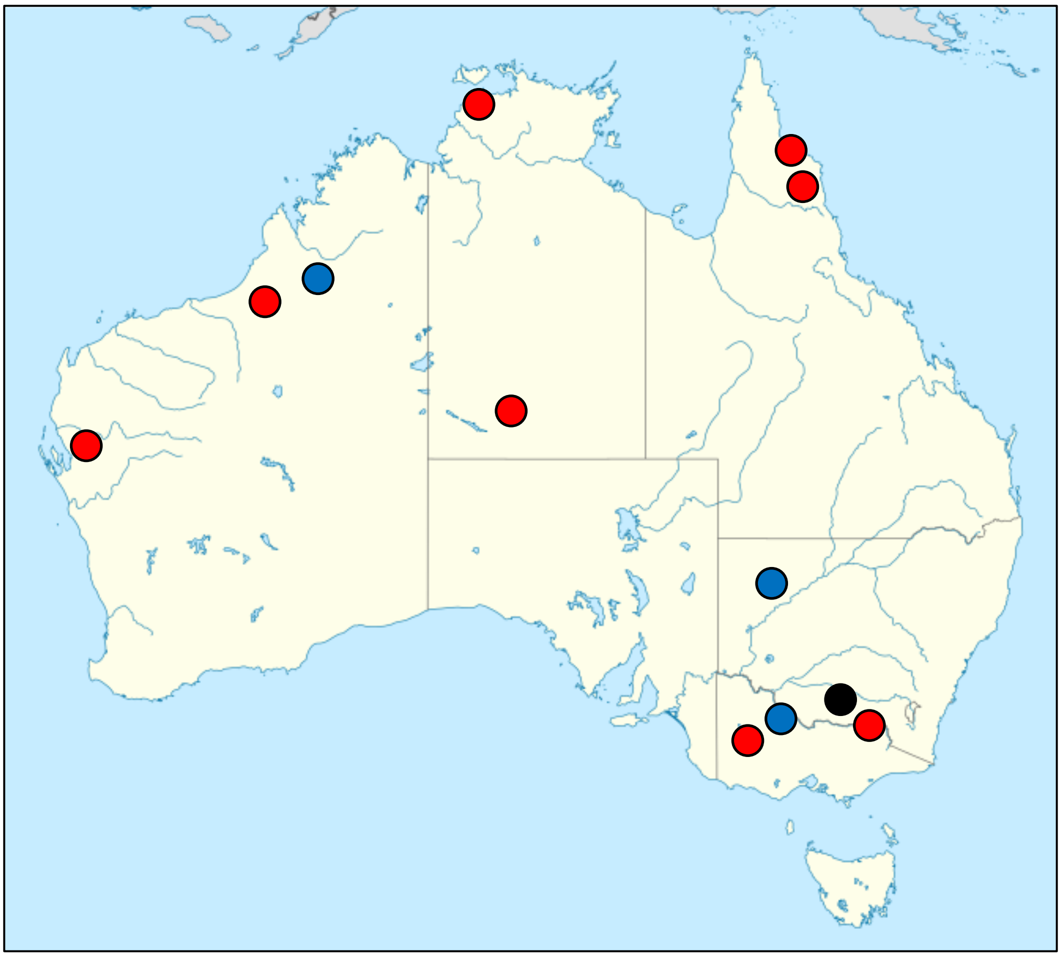

This study

Zhang, et al (2020)

Admixed – not included

Note: One of the samples from Southeast Australia (blue circle in the bottom most corner) was obtained from captivity.

See Table S1 for locations and S2 for details of the samples and genomes.

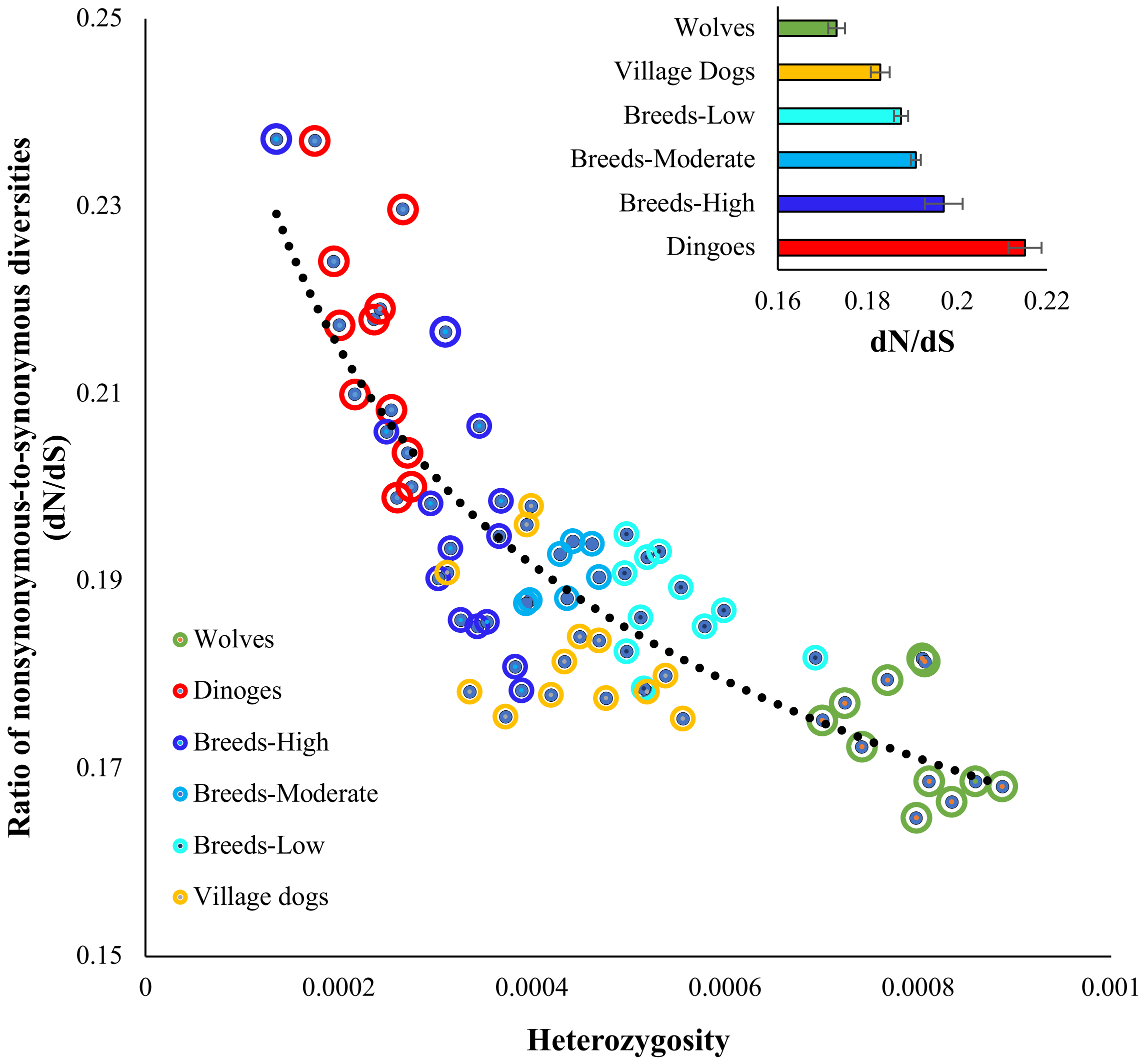

Figure S2. Correlation between the genomic diversity and the ratio of nonsynonymous-to-synonymous diversities (dN/dS) estimated using the dingo reference sequence. The relationship is highly significant (r = 0.59 and P < 0.00001). A regression analysis was used, and the best-fitting Power Law (log-log) curve is shown. **Inset**: Average dN/dS computed for various canine groups. Error bars show the standard error of the mean.

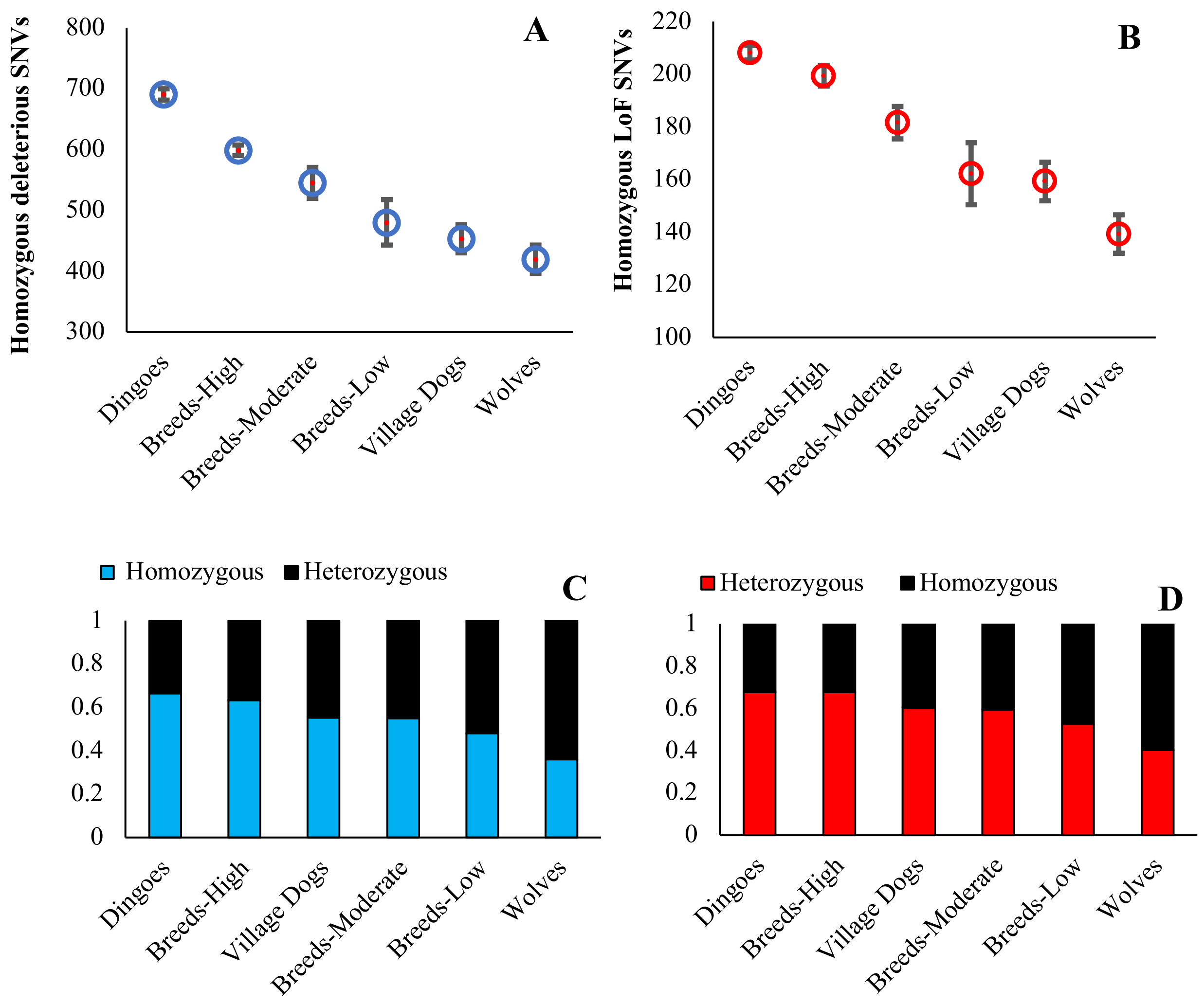

Figure S3. Mean counts of deleterious SNVs **(A)** and loss of function SNVs **(B)** computed for the canine genomes using Dingo reference sequence. Error bars show the standard error of the mean. The stacked columns show the proportion of mean homozygous and heterozygous SNVs using Dingo reference sequence. **(C)** Deleterious SNVs **(D)** Loss of function SNVs.

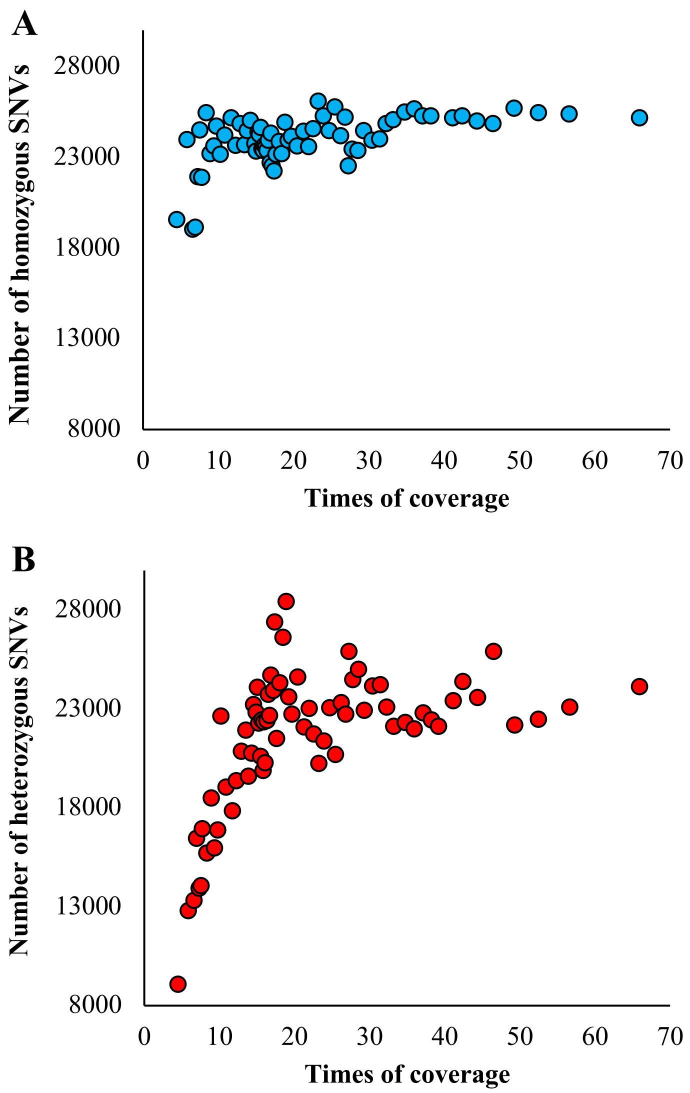

Figure S4: Relationship between the level or depth of sequencing coverage and the number of SNVs called. **(A)** Homozygous SNVs **(B)** Heterozygous SNVs. The number of SNVs remained largely the same after 7.5X coverage for homozygous SNVs and 15X coverage for heterozygous coverage.

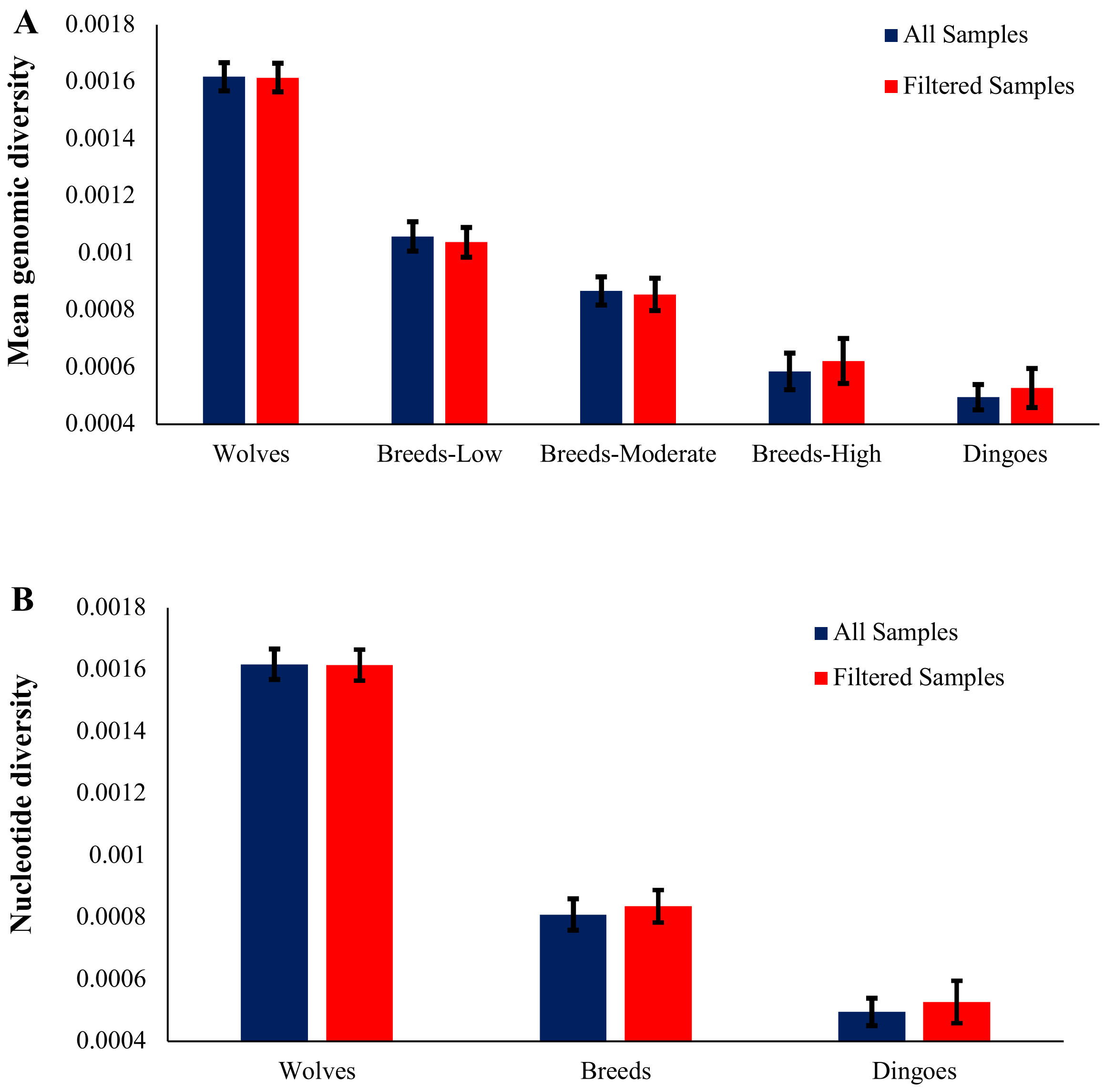

Figure S5: Nucleotide diversities computed for the canine groups before and after genome filtering. Blue bar represents all samples and red bar represents filtered samples (sequencing coverage >= 15X). Error bars show the standard error of the mean. A) Breed dog groups separated based on the level of inbreeding B) All breed dogs combined. All differences between the estimates obtained before and after filtering were not statistically significant.

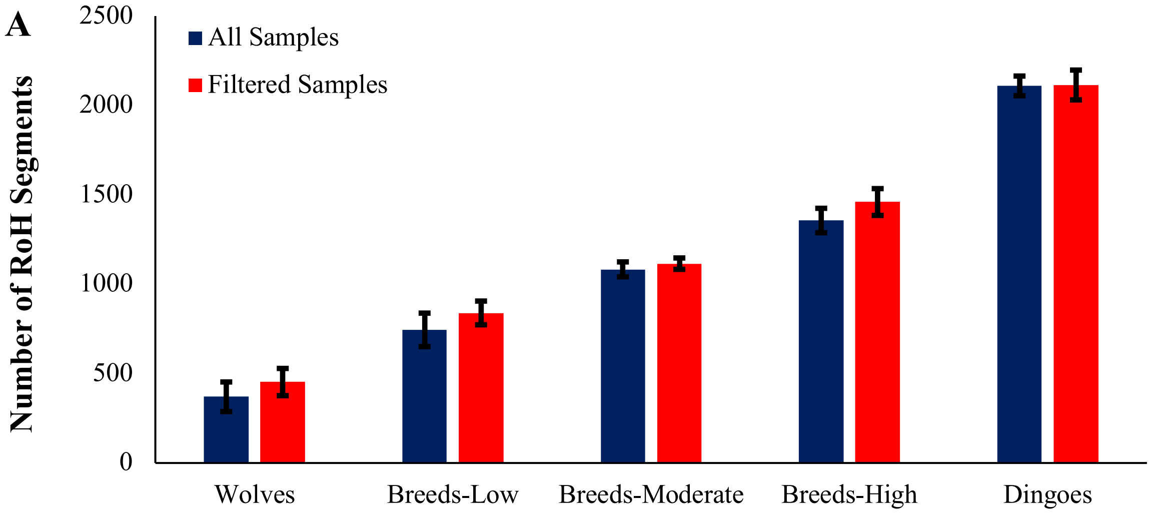

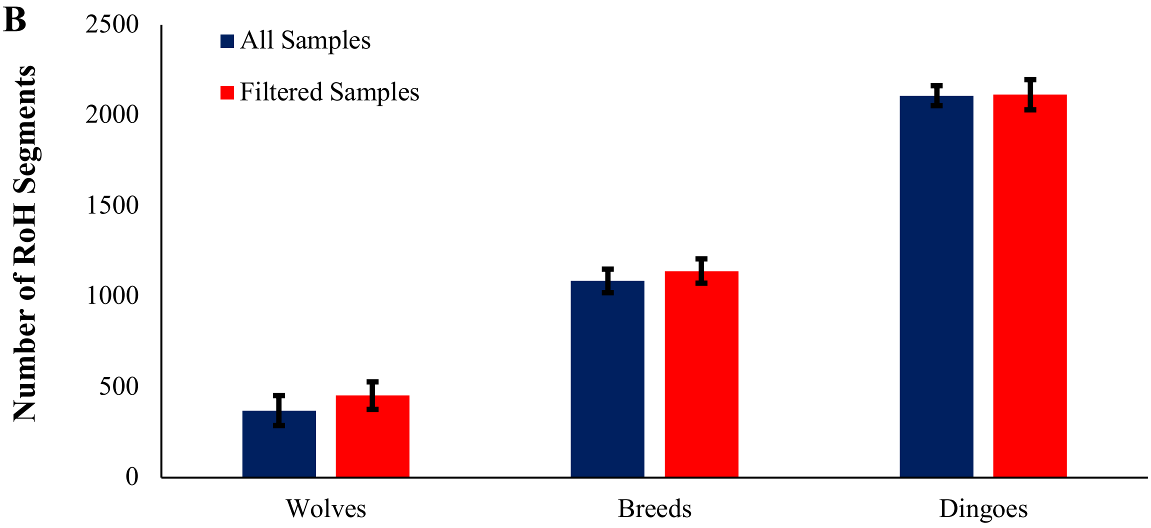

Figure S6. Runs of homozygosity (RoH) tracts estimated for the canine groups before and after genome filtering. Blue bar represents all samples and red bar represents filtered samples (sequencing coverage >= 15X). Error bars show the standard error of the mean. A) Breed dog groups separated based on the level of inbreeding B) All breed dogs combined. All differences between the estimates obtained before and after filtering were not statistically significant.

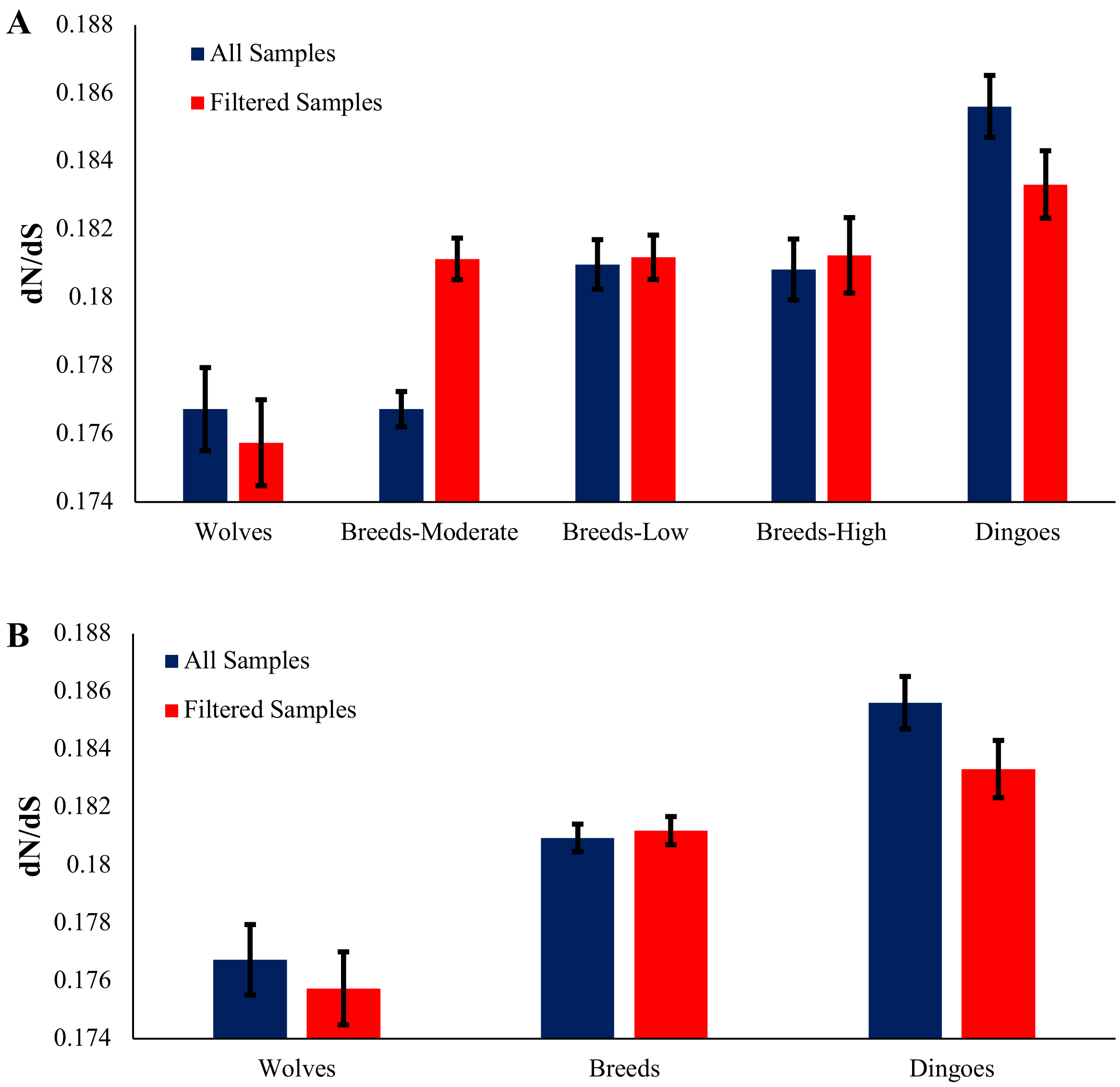

Figure S7: The mean ratio of nonsynonymous-to-synonymous diversities estimated for the canine groups before and after genome filtering. Blue bar represents all samples and red bar represents filtered samples (sequencing coverage >= 15X). Error bars show the standard error of the mean. A) Breed dog groups separated based on the level of inbreeding B) All breed dogs combined. All differences between the estimates obtained before and after filtering were not statistically significant except for the moderate bred dogs.

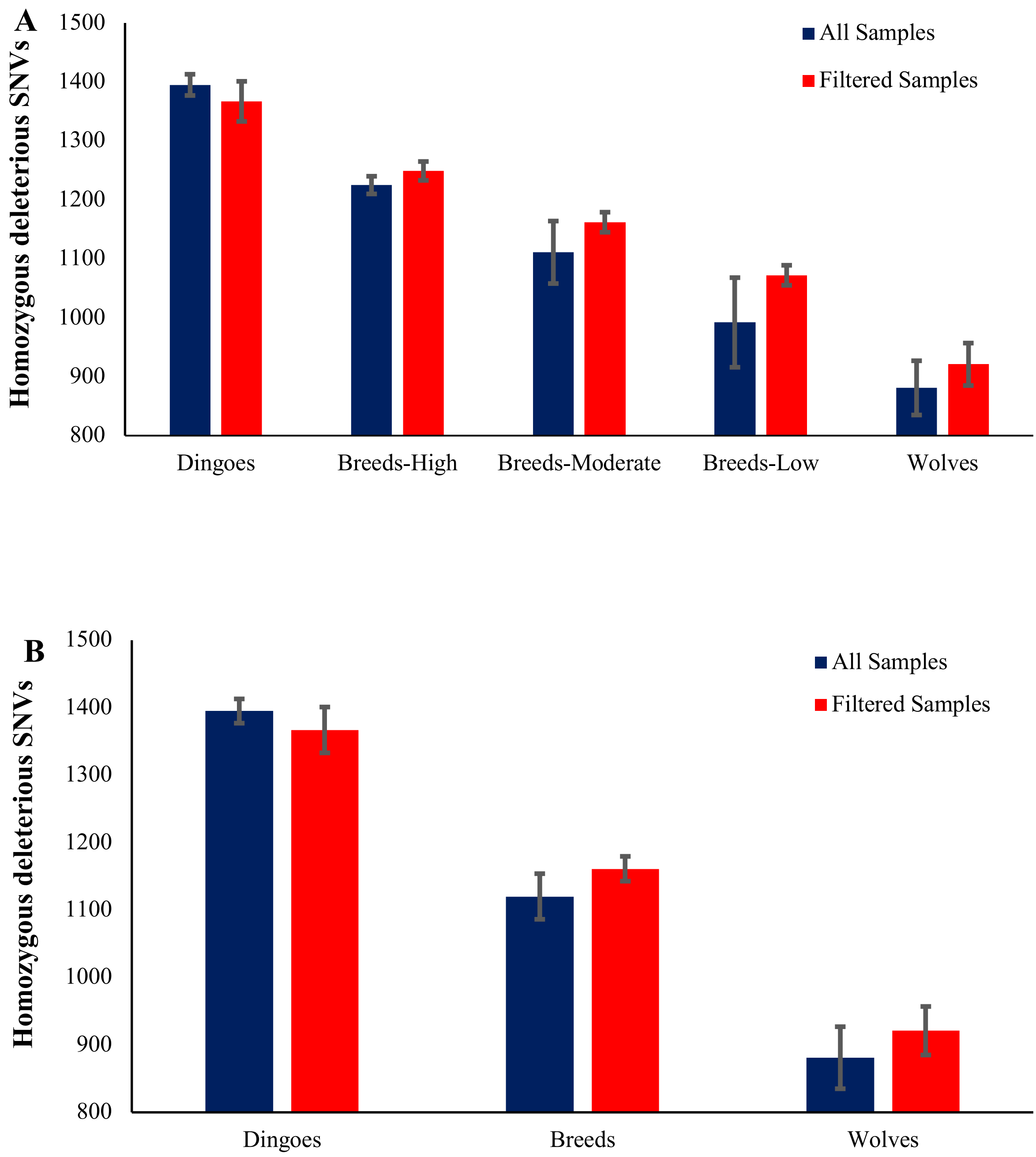

Figure S8: The mean counts of homozygous deleterious SNVs estimated for the canine groups before and after genome filtering. Blue bar represents all samples and red bar represents filtered samples (sequencing coverage >= 15X). Error bars show the standard error of the mean. A) Breed dog groups separated based on the level of inbreeding B) All breed dogs combined. All differences between the estimates obtained before and after filtering were not statistically significant.

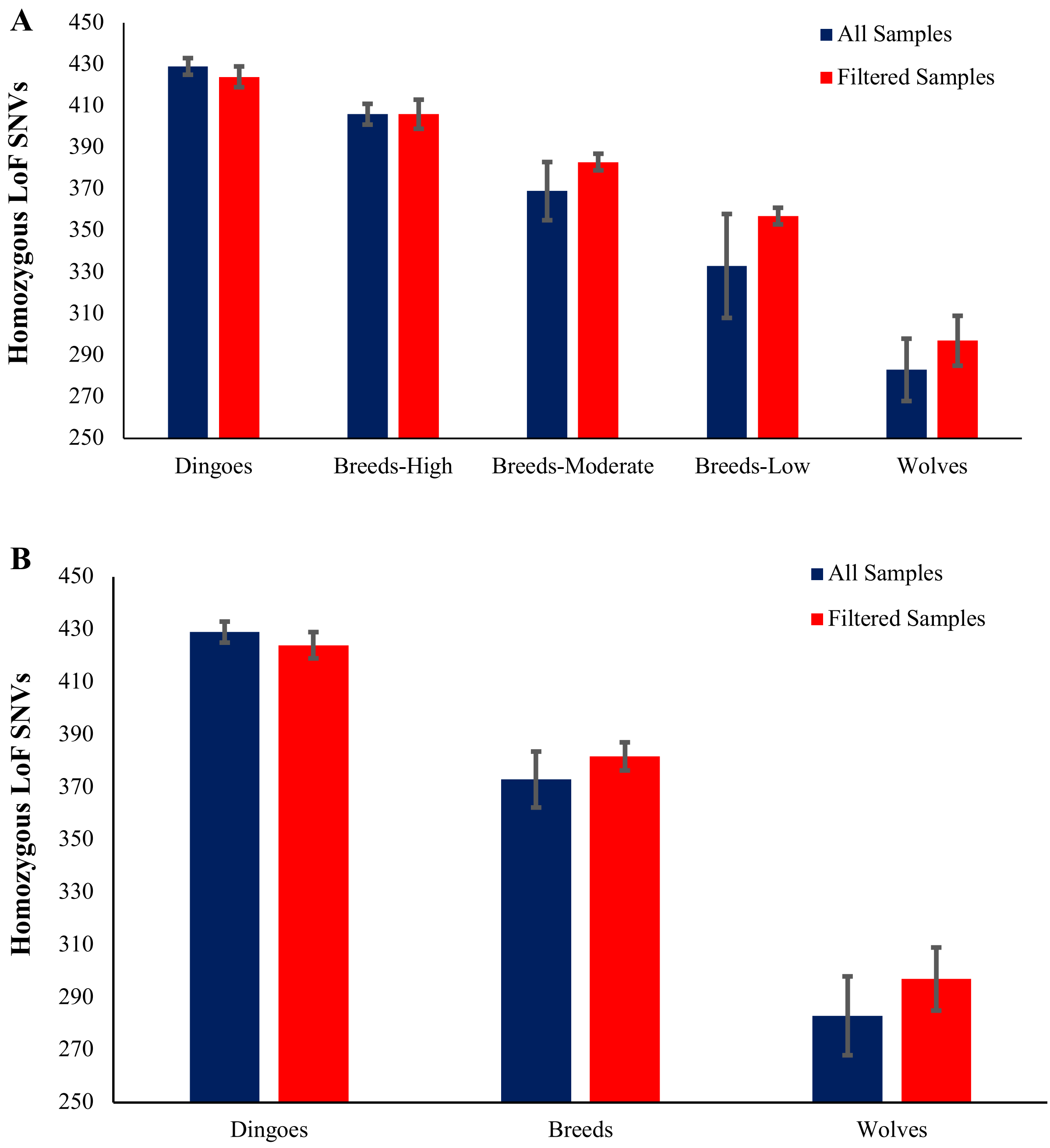

Figure S9: The mean counts of homozygous Loss of Function (LoF) SNVs estimated for the canine groups before and after genome filtering. Blue bar represents all samples and red bar represents filtered samples (sequencing coverage >= 15X). Error bars show the standard error of the mean. A) Breed dog groups separated based on the level of inbreeding B) All breed dogs combined. All differences between the estimates obtained before and after filtering were not statistically significant.

Table S1: Samples location of the whole genomes of canines used in this study

Sample ID	Latitude, Longitude	State
Ernie	-32.09196, 141.4102	New South Wales
Kimmi	-16.86307, 124.4301	Western Australia
Mikey	-37.4524, 144.589	Victoria

Table S2: Sequence Read Archive (SRA) accession numbers of the whole genomes of canines used in this study.

SRA ID	Cover age	Canine Population Name	Breed Name	Reference/ SRA ID
SRR7107643	17	Breeds-High	Norwegian Lundehund	[12]
SRR7120152	40	Breeds-High	Bull Terrier	[9]
SRR7107885	19	Breeds-High	Basenji	[5]
SRR7107902	10	Breeds-High	Soft Coated Wheaten Terrier	[5]
SRR7107968	9	Breeds-High	Border Terrier	[5]
SRR7120213	19	Breeds-High	Scottish Terrier	[9]
SRR7107598	9	Breeds-High	Entlebucher Sennenhund	[2]
SRR7107922	16	Breeds-High	Airedale Terrier	[5]
SRR7107634	12	Breeds-High	Weimaraner	[2]
SRR7107973	33	Breeds-High	Berger Picard	[5]
SRR7120170	38	Breeds-High	Irish Water Spaniel	[9]
SRR7107963	28	Breeds-High	Field Spaniel	[5]
SRR7107867	7	Breeds-High	Australian Cattle Dog	[5]
SRR7107883	20	Breeds-High	English Springer Spaniel	[5]
SRR7107578	25	Breeds-Low	Border Collie	[7]
SRR7107891	19	Breeds-Low	Labrador Retriever	[5]
SRR2094392	9	Breeds-Low	Chow Chow	[4]
SRR7107916	20	Breeds-Low	Yorkshire Terrier	[6]
SRR7107838	11	Breeds-Low	Peruvian Inca Orchid	[3]
SRR7107992	31	Breeds-Low	Alaskan Malamute	[9]
SRR7107895	18	Breeds-Low	Tibetan Terrier	[5]
SRR2095503	48	Breeds-Low	Saluki	[4]
SRR2095478	38	Breeds-Low	Chihuahua	[4]
SRR2095539	48	Breeds-Low	Siberian Husky	[4]
SRR7120156	29	Breeds-Low	Carolina Dog	[9]

SRR7107795	19	Breeds-Moderate	Greyhound	[12]
SRR7107657	18	Breeds-Moderate	Afghan Hound	[11]
SRR7107839	13	Breeds-Moderate	Swedish Lapphund	[3]
SRR7107884	20	Breeds-Moderate	German Shepherd Dog	[5]
SRR7107933	21	Breeds-Moderate	Shiba Inu	[5]
SRR5664959	30	Breeds-Moderate	Cocker Spaniel (American)	[8]
SRR7120187	29	Breeds-Moderate	Miniature Poodle	[9]
SRR7107676	4	Village Dog	Village Dog - Egypt	[1]
SRR7107649	5	Village Dog	Village Dog - China	[12]
SRR2095463	12	Village Dog	Village Dog – Bernese Mountain Dog	[4]
SRR7107690	6	Village Dog	Village Dog - Lebanon	[1]
SRR7107703	6	Village Dog	Village Dog - Vietnam	[1]
SRR7107828	8	Village Dog	Nigerian Indigenous Dog	[3]
SRR7107684	9	Village Dog	Village Dog - India	[1]
SRR7107700	9	Village Dog	Village Dog - Qatar	[1]
SRR7107697	9	Village Dog	Village Dog – Papua New Guinea	[1]
SRR7107693	9	Village Dog	Village Dog - Namibia	[1]
SRR7107698	12	Village Dog	Village Dog - Portugal	[1]
SRR7107823	11	Village Dog	Vietnamese Indigenous Dog	[3]
SRR7107702	11	Village Dog	Village Dog - Taiwan	[1]
SRR7107786	22	Wolf	Wolf	[10]
SRR7107910	20	Wolf	Wolf	[9]
SRR7107787	20	Wolf	Wolf	[10]

SRR7107542	14	Wolf	Wolf	[2]
SRR7107909	16	Wolf	Wolf	[9]
SRR7107540	18	Wolf	Wolf	[2]
SRR7107783	21	Wolf	Wolf	[10]
SRR7107776	13	Wolf	Wolf	[10]
SRR7107778	22	Wolf	Wolf	[10]
SRR7107987	28	Wolf	Wolf	PRJNA361023
SRR7107777	22	Wolf	Wolf	[10]
SRX7276152	10	Dingo	Fraser Dingo	[13]
SRX7276159	11	Dingo	Dingo-NW	[13]
SRX7276160	16	Dingo	Dingo-NW	[13]
SRX7276154	15	Dingo	Dingo-NW	[13]
SRX7276158	13	Dingo	Dingo-NW	[13]
SRX7276153	15	Dingo	Dingo-NW	[13]
SRX7276156	15	Dingo	Dingo-NW	[13]
Ernie	8	Dingo	Dingo-NW	This study
Kimmi	10	Dingo	Dingo-NW	This study
Mikey	10	Dingo	Dingo-SE	This study
SRX7276151	17	Dingo	Dingo-SE	[13]
SRX7276157	7	Dingo	Dingo-SE	[13]
SRX7276155	9	Dingo	Dingo-SE	[13]

References

1. Auton, A., et al., Genetic recombination is targeted towards gene promoter regions in dogs. PLoS Genet, 2013. 9(12): p. e1003984.

2. Bauer, A., et al., A de novo variant in the ASPRV1 gene in a dog with ichthyosis. PLoS genetics, 2017. 13(3): p. e1006651-e1006651.

3. Becker, D., et al., A GJA9 frameshift variant is associated with polyneuropathy in Leonberger dogs. BMC genomics, 2017. 18(1): p. 662-662.

4. Decker, B., et al., Comparison against 186 canid whole-genome sequences reveals survival strategies of an ancient clonally transmissible canine tumor. Genome research, 2015. 25(11): p. 1646-1655.

5. Dreger, D.L., et al., Commonalities in Development of Pure Breeds and Population Isolates Revealed in the Genome of the Sardinian Fonni's Dog. Genetics, 2016. 204(2): p. 737-755.

6. Dutrow, E.V., J.A. Serpell, and E.A. Ostrander, Domestic dog lineages reveal genetic drivers of behavioral diversification. Cell, 2022. 185(25): p. 4737-4755.e18.

7. Forman, O.P., et al., An Inversion Disrupting FAM134B Is Associated with Sensory Neuropathy in the Border Collie Dog Breed. G3 (Bethesda, Md.), 2016. 6(9): p. 2687-2692.

8. Kim, J., et al., Genetic selection of athletic success in sport-hunting dogs. Proceedings of the National Academy of Sciences of the United States of America, 2018. 115(30): p. E7212-E7221.

9. Plassais, J., et al., Whole genome sequencing of canids reveals genomic regions under selection and variants influencing morphology. Nature communications, 2019. 10(1): p. 1489-1489.

10. Ramos-Madrigal, J., et al., Genomes of Pleistocene Siberian Wolves Uncover Multiple Extinct Wolf Lineages. Current biology : CB, 2021. 31(1): p. 198-206.e8.

11. Serres-Armero, A., et al., Copy number variation underlies complex phenotypes in domestic dog breeds and other canids. Genome research, 2021. 31(5): p. 762-774.

12. Tonomura, N., et al., Genome-wide association study identifies shared risk loci common to two malignancies in golden retrievers. PLoS genetics, 2015. 11(2): p. e1004922-e1004922.

13. Zhang, S.-J., et al., Genomic regions under selection in the feralization of the dingoes. Nature communications, 2020. 11(1): p. 671-671.
